# A user-centric framework for harmonizing scientific name usage

**DOI:** 10.3897/BDJ.14.e184830

**Published:** 2026-03-27

**Authors:** Walter G. Berendsohn, Olaf Bánki, Markus Döring, David Fichtmueller, Anton Güntsch, Roger Hyam, Sofie Meeus, Andreas Müller, Bart Vanhoorne

**Affiliations:** 1 Freie Universität Berlin, Botanic Garden and Botanical Museum, Berlin, Germany Freie Universität Berlin, Botanic Garden and Botanical Museum Berlin Germany; 2 Catalogue of Life, Amsterdam, Netherlands Catalogue of Life Amsterdam Netherlands; 3 GBIF, Copenhagen, Denmark GBIF Copenhagen Denmark; 4 Royal Botanic Garden Edinburgh, Edinburgh, United Kingdom Royal Botanic Garden Edinburgh Edinburgh United Kingdom; 5 Meise Botanic Garden, Meise, Belgium Meise Botanic Garden Meise Belgium; 6 VLIZ, Oostende, Belgium VLIZ Oostende Belgium

**Keywords:** name matching, name-matching services, taxonomic databases, data linkage, taxonomic datasets, taxonomic aggregators, taxonomy, biodiversity research

## Abstract

Biodiversity data linkage is a prerequisite for addressing pressing societal and research questions. Digitisation of resources has progressed rapidly, but this has led to a number of parallel approaches for datasets and services, making it difficult for users to identify the appropriate tool for their requirements. While scientific names are a key element for data linkage, they are not fully suited to this role. Many of them are not unique identifiers for taxa (such as the species that form the basic building block of a large part of biodiversity information) because taxonomic research has resulted in name changes. Likewise, usage is prone to errors like misspellings or misapplication of nomenclatural rules. This complexity and unreliability may demotivate data providers from readying their valuable datasets for integration into an interlinked landscape of biodiversity information. Correct linking of scientific names is an important element in this process of linking data from various sources. Aggregated taxonomic datasets and name-matching services are an essential infrastructure to build tools for this purpose and are increasingly using unique, resolvable and persistent name identifiers. Accessing the aggregated dataset via these identifiers can also display connections between the names within the dataset, linking, orthographic variants to correct spelling, synonyms to accepted taxon name etc. External datasets that hold valuable research and usage information as taxon-associated data can also be linked directly via these identifiers. This helps them to meet the FAIR data criteria (Findable, Accessible, Interoperable, Reusable). Name matching finds the aggregator's name identifier, adding this identifier to the local data record may help to further interconnect biodiversity information, if data providers are motivated to partake in this process of enabling data linkage. We posit that this motivation can be increased by simplifying the process of choosing the right method for their specific needs, which may vary widely. We thus propose a framework that helps users to select the optimal workflow to address these needs. This involves: (i) identifying and categorising the specific criteria that characterise different user needs; (ii) based on these criteria, defining the necessary metadata both for taxonomic datasets accessed by name-matching services and the name-matching services themselves; (iii) providing tools based on these metadata that will allow users to interactively and confidently choose the best-fit target dataset and name matching service for their specific purpose.

## Background

The work presented here was carried out within the TETTRIs (Transforming European Taxonomy through Training, Research and Innovations) project, with the primary objective of furthering the "integration of taxonomic resources to enable cross-domain data linkage" (TETTRIs 2022), a process that we consider a central enabler for knowledge transfer and creation in biodiversity sciences.

### Biodiversity data linkage

Data linkage (or record linkage, Harron (2016)) is "the aggregation of data from different sources concerning the same entity or individual" (Vaccaro and Swerts 2022). In our context, this entity is the taxon: a group of organisms sharing defined characteristics and distinguishable from other groups. Taxonomists assign names to these groups following internationally accepted rules. Taxa are generally arranged in hierarchical ranks that reflect presumed evolutionary relationships. Amongst these ranks, the species-level is most relevant in applied contexts, because species are generally regarded as concrete biological entities that can be used, are endangered, are found in a garden, possess defined ecological traits etc. Diverse data sources contain information related to the same taxa and publication as open data under open data licences is becoming the norm. In the process of data linkage, knowledge from disparate sources is brought together to characterise taxa and their interaction with themselves and with the environment (and humans). These taxa are typically identified by scientific names, though molecular sequences are increasingly used as proxies. The "Codes of Nomenclature" (ICZN 2012, Parker et al. 2019, ICTV 2024, Turland et al. 2025) provide strict rules as to the syntax of scientific names as well as requirements for their publication; animals, fungi and plants not only have to be described, but the published name has also to be explicitly tied to a single physical item, the so-called nomenclatural type (mostly a specimen). Traditionally, other information is then linked by using the taxon name as a common identifier. A practical approach to achieving this linkage for digital data is to map locally used taxon names *(local data)* to the names present in taxonomic aggregator databases (but see caveats pointed out below). Here, *local data* encompasses everything from individual researchers’ spreadsheets - such as those used in ecological studies - to national or supranational taxonomic databases. The latter may, in turn, also serve as taxonomic aggregators and targets for name matching in a local setting (see Table 1 for definitions of terms used).

### Taxonomic aggregators

Attempts to provide global checklists of organisms go back to early taxonomists like Linnaeus who were able to provide more or less comprehensive checklists of the species known to western science in the mid-18^th^ century. The knowledge base soon exploded and later attempts were reduced to trying to cope with the publication of new names, achieved at least in some groups such as plants (e.g. the Index Kewensis, Lughadha (2004)). With the advent of computers and networking technologies, the vision of a global checklist of organisms (and their *scientific names*) seemed to become realistic again, but from early attempts (Bisby et al. 1994) to today, it has proven a rather difficult task that is by no means finished. In the last three decades, much progress has been made by increasing the number of digitally available described taxa (Bánki et al. 2025, WoRMS Editorial Board 2025). Still, several organism groups have data gaps, at times at both national and international scales and may even lack the necessary taxonomic experts all together (Hobern et al. 2021). Aggregating taxonomic data from diverse sources into unified datasets facilitates knowledge transfer and enables new research opportunities. For example, the specimen and observation data aggregated by the Global Biodiversity Information Facility (Heberling et al. 2021, GBIF Secretariat 2024) and the taxonomic literature aggregated by the Biodiversity Heritage Library (BHL 2007) are now indispensable tools for taxonomy and biodiversity research in general. For the names of taxa and (less controversially) scientific organism names in general, much progress has been made in the recent past. The existing nomenclatural and taxonomic online lists are heavily used. In some communities, taxonomic institutions are becoming involved in organising taxonomists to accelerate these developments; the World Flora Online (WFO) initiative with its WFO Plant List (2021-) assembled by Taxonomic Expert Networks (TENs) is a good example. The World Register of Marine Organisms (WoRMS Editorial Board 2025) sets an example for an organisation with an institution providing the organisational and technical base for a large-scale peer-level cooperation. Generally, Lien et al. (2023) found a “high degree of agreement on the need for a global list of accepted species and their names” as the results of their global survey of taxonomists, scientists from other disciplines and users of taxonomy. What is lacking is the financial support to build long-term sustainable infrastructures to support such development and maintain the momentum.

### A way forward

As a result, we think that *local users* who are motivated to improve their taxonomic data and search for on-line resources to do so are faced with a confusing picture, especially when working with a broad range of *taxonomic groups*. As Garnett et al. (2020) put it: "users, who may lack relevant taxonomic expertise, [are forced] to choose between competing lists and taxonomic treatments of groups even though they rarely understand the rationale why competing lists exist and are confused by the differences between alternate taxonomic concepts and classifications". We will here try to show ways how existing infrastructures could be used to help *local users* increase the quality of their *scientific name* data and, thus, enable linking their information by means of the names. In the following, we first have to point out some inherent problems associated with the use of *scientific names* for data linkage. We will then have a closer look at the requirements on the user side, to go on to existing *aggregators* and *name-matching services*. From that, we will suggest a number of criteria for tool selection on the user’s side and propose *metadata* elements that will provide the necessary information for *taxonomic datasets* and *name-matching services*. As a proof of concept, a demonstrator application for an updatable and interactive guide to solutions for individual use cases is presented.

## Material and methods

We used systematic web searches and followed citations in literature to identify, test and document 20 *name-matching services* and 34 *taxonomic datasets* (56 dataset–service combinations - some services refer to a single *taxonomic dataset*, some allow to access several held in a repository). To ensure consistency, key terms used throughout this article are defined in Table 1 and written in italics throughout the article.

As a contribution towards a formally defined common API (Application Programming Interface) for *name-matching services*, we compiled and compared the input parameters accepted by existing *name-matching services* and the fields they return. From this inventory, we proposed a set of common input parameters and output fields with standardised names (see Hyam et al. (2025a) for details).

In the context of ongoing work, we extensively used botanical datasets (as *local data*) to test *name-matching services*. These included Euro+Med PlantBase (Euro+Med 2006-), which contains more than 150,000 names and herbarium records from a major herbarium (Madrid) and from smaller collections managed through the JACQ system (Rainer et al. 2023). We also used the names collected by the Global Biodiversity Information Facility (GBIF) for botanical occurrence records (Berendsohn et al. 2024b). In addition, several datasets were matched against botanical *taxonomic datasets* when preparing them as a contribution to the WFO Plant List, inter alia datasets from the Caryophyllales network (Korotkova 2022) and the Cichorieae dataset (Kilian and Raab-Straube 2009-). A use case spanning all organism groups was developed in collaboration with the German NFDI4Biodiversity network (Ebert et al. 2023) using long-term ecological research datasets from LTER Germany (Frenzel 2025). Zoological datasets for testing includes those obtained from Catalogue of Life ChecklistBank (Bánki et al. 2023), from the GBIF and, for example, the German freshwater fish monitoring dataset (Friedrichs-Manthey et al. 2024). This type of “taxonomic annotation” of ecological data was also addressed in detail by Islam et al. (2024) in their comprehensive case study, which highlighted the difficulties faced by non-taxonomists when performing such tasks. The potential user requirements set out in the current article were derived from Islam et al., from communications with *local users* providing data for testing and from our own experience as *local users* of *name-matching services.* The process was further informed by discussions held in project meetings and at international congresses (see, for example, Berendsohn (2023a), Berendsohn (2024), Anonymous (2024a)).

We did not use procaryotic or viral names for testing, but identified major data sources (see Appendix 1).

## Inherent caveats of the name-matching workflow

### *Scientific names* as unique identifiers

Using a *scientific name* as the identifier of a taxon is a pragmatic approach to link cross-domain biodiversity data. The names provide a standard way to connect information about a given taxon, for example, a species. Thus, data from many different sources can be combined. For example, information on the species' uses (e.g. medicinal, cultural, construction), its morphology (appearance and form), its geographical distribution, its conservation status (e.g. threatened by extinction) and threats it poses (poison, invasive, pest). Such data have been collected by humans for millennia and recorded in persistent forms for centuries. Since the mid-1700s, this has been in connection with *scientific names* governed by internationally agreed rules.

However, the approach entails several notable caveats. A common quality problem in the data themselves is mis-identification: an organism may be labelled with a name that represents a different taxon. Furthermore, as Hyam et al. (2025a) point out, homonyms and isonyms can lead to false positive matches; non-matches can be caused by variations in the orthography of the *canonical name* or (often!) in the author/year string, which may arise from changes in the rules, human error, transliteration or OCR mistakes; and names found in recent literature or databases may be the result of generative AI hallucinations (Hyam 2024), a phenomenon that can undermine efforts to build dependable taxonomic datasets.

Beyond these issues, *scientific names* present inherent issues that can affect the quality of *data linkages*: the international rules of nomenclature that govern *scientific names* can require that the name of a taxon be changed. Such nomenclatural name changes can be handled automatically if a *taxonomic dataset* encompasses the necessary information on the nomenclatural and taxonomic relationships of the name in question. However, the rules may also result in the same name referring to taxa differently circumscribed at different times or by different experts. This is where the concept problem arises, which we have to address in some detail before returning to the core subject of name matching.

### The concept problem

A far more complex issue is that *scientific names* are essentially pointers to a concept of a taxon, for example, that of a species. These concepts are scientific hypotheses about the descent and boundaries of the taxon and, as new research on species evolution accumulates, hypotheses may change. This means that taxa formerly treated as separate may be merged, rendering some names synonyms; conversely, a taxon long regarded as single may be split into multiple taxa following careful investigation.

Every synonym should refer to the accepted name of a taxon. However, because names are not necessarily changed when a taxon’s circumscription changes (following the rules of nomenclature), taxonomic expertise is sometimes required to decide whether data provided under a particular taxon name from different sources actually refer to the same currently accepted taxon. To complicate matters further, the same name can sometimes refer to different accepted taxa because the naming occurred under different codes of nomenclature (see Checking the names of cyanobacteria in Appendix 1). “*In practice, many people provide taxonomic names in their datasets or publications but not a source specifying a usage. The information needed to map the relationships between names and usages in taxonomic monographs or revisions is typically not presented in a machine-readable format*” (Sterner et al. 2020). Remsen (2016) provides a thorough analysis and calls for “*comprehensive taxonomic thesauri ... to model the relationships between names and taxa*” to come to grips with it. This gets to the core of the problem. Attempts have been made to address it by distinguishing taxon concepts and labelling them with non-nomenclatural identifiers. However, applying this approach to all or large groups of organisms would require an enormous expansion of the knowledge base. Consequently, such data currently exist only for a few model datasets and, “*in practice, aggregators have made fundamental design choices […] by excluding taxonomic conflict as an aspect of biodiversity knowledge worthy of being preserved*” (Franz and Sterner 2018). Sterner et al. (2020) also note that “*New approaches are making progress on these obstacles. Theoretical advances in the representation of taxonomic intelligence have made it increasingly possible to implement efficient querying and reasoning methods on name-usage relationships* (Chen et al. 2014, *Franz et al. 2015*, Chawuthai et al. 2016). *Perhaps most importantly, growing efforts to produce name-usage mappings on a medium scale by data providers and taxonomic authorities suggest an all-or-nothing approach is not required”.* We agree with the latter statement, but view the progress so far as not usable in real-world contexts.

A more practical approach may be to enable communication of changes once a linkage between a set of *local data* and an aggregator's *taxonomic dataset* has been established by name matching and subsequent storage of aggregator *name IDs* locally. The idea of a "Concept subscription service" (Berendsohn 2024) was developed into a technical specification of a "Taxonomic activity notification service" by Hyam et al. (2025b) and could be implemented as an additional aggregator service. This requires further research into the categories of changes in the content of taxonomic databases that influence the taxonomic concept behind a *scientific name* (see TETTRIs (2024b)). However, a functional workflow for name matching remains a prerequisite for using such a service and, to be truly useful, it depends on the availability of comprehensive datasets covering names across all *taxonomic groups*.

### Proliferation of identifiers

**Figure 1.**
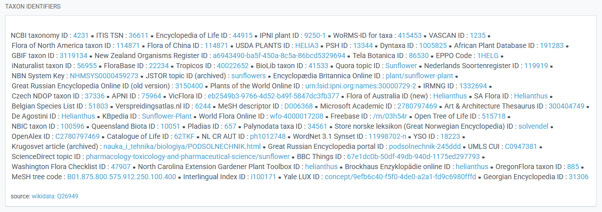
“Taxon Identifiers”. Source: *Helianthus* L. in GBIF Secretariat (2023). GBIF Backbone Taxonomy. Checklist dataset https://doi.org/10.15468/39omei accessed via GBIF.org on 28-09-2025 (Source of data: Wikidata https://www.wikidata.org/wiki/Q26949).

While name matching and subsequent correction or harmonisation of names can help to link data via the name strings themselves, data integration across biodiversity information systems becomes much more efficient when persistent *name IDs* are used (Rambold et al. 2017, but see caveats below). To serve that function, clear rules and transparent policies for assigning identifiers to name records are essential. The current situation, however, is marked by a proliferation of so-called “taxon identifiers”: for example, GBIF lists 64 different “taxon identifiers” for the sunflower genus *(Helianthus)* citing Wikidata (Fig. 1). Taxonomic communities are, therefore, urged to ensure the availability of well-documented truly stable *name identifiers* in their domain, independent of the taxonomic acceptance of the individual name. This also enables machine interoperability and forms an important step towards the shift to a data engineering approach as deemed essential for a successful global data infrastructure, for example, by the WorldFAIR project (Hodson and Gregory 2024).

This proliferation raises practical concerns for users who must, or choose to, add *name IDs* to their data during “taxonomic annotation”. A common question is how stable these identifiers really are in practice: “*Can my sunflower turn into a bug tomorrow*?” (F. Zander, pers. comm.) - i.e may the found *name ID* be reused for another name at a later stage? Part of the confusion stems from conflating *name IDs* and taxon identifiers. A name is governed by the Codes of Nomenclature and is, therefore, comparatively stable; a taxon is a conceptual entity not governed by such codes, making its identifiers intrinsically less stable (see the taxon concept discussion above) and the actual name the taxon identifier resolves to may change, pointing to the accepted *scientific name* for the taxon. Consequently, using the term “taxonID” for name records is potentially misleading, since taxa are always associated with an accepted name. However, this term has become thoroughly established by its use in standards like Darwin Core (Wieczorek et al. 2012) and should, thus, not be touched.

The need for durable, unique and resolvable *name IDs* arises from the requirement to verify how a given name is treated in aggregated datasets. Ideally, identifiers should resolve names (including synonyms, but also orthographic variants, isonyms and other nomenclaturally invalid designations) both to their nomenclatural background (e.g. correct spelling) and to their taxonomic usage (i.e. current accepted name). *Nomenclators* and authoritative name lists are best placed to provide such *name IDs*, but even these rarely publish explicit policies on how identifiers behave in cases of name changes, orthographic corrections, authorship revisions or adjustments to nomenclatural citations. Few *taxonomic datasets* make such policies transparent (see TETTRIs (2025)).

### The illusion of precision

Linked Data methods can improve data precision, but if the underlying semantic resources are unstable or inconsistent, they may reduce data quality and re-usability. Scientific names are not fixed references to clearly defined taxonomic concepts. Converting names in source data into persistent, resolvable identifiers tied to a particular taxonomic dataset can create a false sense of accuracy. In practice, this often remains a time-bound, deterministic mapping of a text string to a presumed concept defined within that dataset. During this process, noise and errors may be smoothed away, leaving future users with unwarranted confidence in the results. What follows from this? First, *local users* should treat name-matching as part of their research workflow and apply it critically, following good scientific practice, especially when using fuzzy matching (Grenié et al. 2022). *Local users* that are actually aggregators of research data (such as repositories of ecological data) should preserve original, unmodified source data after applying such services, so that matching can be repeated for specific purposes. There are actually two issues here - depending on the policies of the responsibles for the *taxonomic aggregator, scientific names* may be modified in the *taxonomic dataset*, so that, for example, the previously matched names now match differently. A second issue (not really focused on in the context of this article) are changes in the taxonomy, i.e. what accepted name or higher taxon in the aggregator's classification the matched name (or its *name-ID*) now leads to. We hope that our framework supports informed service selection for the core *name matching* process. For *taxonomic aggregators*, this underlines the need for a shared and harmonised strategy for managing *name IDs* and documenting *taxonomic datasets* and *name-matching services*.

## The user side: matching and linking

### Who are (potential) users?

We examined a range of use cases across different research areas. At a basic level, the distinction between name matching and searching can be blurred (see Hyam (2024)). On-line searching is often carried out to obtain taxon-*associated data*, for example, its uses, ecological traits, or conservation status. Such searches are very common. In this article, however, we do not consider searches whose primary aim is retrieving *associated data*. Instead, our goal is to improve linkage of *associated data* by improving name-based data integration. We therefore focus on *local users* who perform name matching to verify correct name spelling, nomenclatural validity, taxonomic acceptance or systematic placement (e.g. family assignment) in the target *taxonomic dataset.* Ideally, this produces a persistent link between the name in the aggregator and the user’s name. Potential users in this context are anyone who creates or works with datasets that include *scientific names.* This spans many disciplines: biodiversity, ecology and environmental sciences; conservation biology; agriculture, forestry and fisheries; marine sciences; geology; public health and epidemiology; forensics; archaeology; legal, commercial and administrative domains; bioinformatics; art; and citizen science. Relevant *local data* may include observation or measurement records (traits, abundances, biomass, successional stages or long-term monitoring series). Included are also lists of labels (taxonomic annotations) for images, molecular sequences, natural-history specimens, sediment or forensic samples or commercial and pharmaceutical products. *Local data* may also document organismal relationships, such as pollination, pest–host interactions, disease associations, food chains or parasitism. Ecosystem assessments, based on indicator species, palaeontological material or historical age determinations, are included as well. Other examples are specialised taxonomic lists (e.g. Red Lists, CITES appendices, Natura 2000 species, SUDs - Special Use Designations) and commercial designations. Core taxonomic resources, such as monographs, checklists, floras, faunas and mycotas, also fall within this scope.

### User motivation

Name matching as a means of *data linkage* requires effort from users.

For those compiling core taxonomic datasets as *local data*, this effort comes naturally, because name matching directly improves their product. Assuming good data quality at the aggregator side, name matching can reveal orthographic errors, improve adherence to standards (e.g. author abbreviation in botanical names), detect homonyms and expose discrepancies in name acceptance for nomenclatural or taxonomic reasons. Differences in taxonomic structure or opinion may be revealed and spark discussion (see, for example, debate on species aggregates in Malekmohammadi et al. (2024)).

Researchers who do not primarily focus on taxonomy may require additional motivation to perform name matching. Encouraging data creators to produce FAIR-compliant results (Wilkinson et al. 2016) should be part of general scientific practice. Motivations for applying name matching can be grouped into two broad types: intrinsic scientific motivation and external or mandated motivation. Intrinsic scientific motivation is driven by the pursuit of high-quality scientific data: ensuring correct name orthography, accurate citation of a taxon’s classification (e.g. family assignment) and, when taxa are the study objects, efficient retrieval and integration of all relevant information, including data linked to synonymous or related names.

External motivation comes from institutional, legal, infrastructural or publishing requirements. Examples include funder-imposed conditions (e.g. BEXIS-2 project requirements in Germany, Chamanara et al. (2021)), name registration for electronic publication in zoology and mycology or regulatory frameworks (e.g. country reporting under the EU Flora Fauna Habitat Directive, EU (1992)). EU-nomen (a.k.a. PESI, de Jong et al. (2015)) is explicitly recommended as a reference list for species names in the technical guidance document on how species distributions under the INSPIRE directive of the EU Commission might be implemented by EU Member States (European Commission 2024). Catalogue of Life (Bánki et al. 2025) is used as the taxonomic reference for the EUNIS system (https://eunis.eea.europa.eu/about), harmonising the species reporting by Member States under the EU Birds, Habitats and Invasive Species Directives and is the taxonomic reference for the Global Biodiversity Information Facility. The World Register of Marine Species (WoRMS Editorial Board 2025) is the taxonomic reference for the Ocean Biodiversity Information System (OBIS, https://obis.org). The World Flora Online (WFO Plant List 2021-) contributes to the Global Plant Conservation Strategy. Some journals already require citing *name IDs*, at least for new organism names, for example, some PLOS journals require the IPNI LSID (Govaerts et al. 2022) for new botanical names. For electronic-only publication of new zoological names, registration and citation of a ZooBank LSID are mandatory (ICZN 2012); for fungal names, all publications must cite the *name ID* issued by one of the recognised repositories (Turland et al. 2025). AlgaeBase (Guiry and Guiry 2025) requires a PhycoBank ID for new names. Journals that support the authoring process are beginning to link *scientific names* automatically and display identifiers — for example, Pensoft's ARPHA writing tool now uses Global Names Recognition and Discovery Services (Mozzherin and Shorthouse 2019) and Catalogue of Life ChecklistBank (Bánki et al. 2023) to obtain identifiers (T. Georgiev and L. Penev, pers. comm.). These requirements can already increase the fairness of research data, since links to persistent identifiers may be added where data are deposited. Some taxonomic databases import those registrations and identifiers semi-automatically (e.g. Catalogue of Life ChecklistBank via Plazi TreatmentBank - Plazi (2021); WoRMS via Pensoft - Van Maldeghem et al. (2022)).

The institutional environment also influences uptake. Where biodiversity informatics research is carried out, FAIR principles are more likely to become part of the research workflow. Today, open data are an essential prerequisite for the work of taxonomists and for biodiversity research in general. Institutions producing taxonomic outputs (revisions, checklists, *nomenclators*) tend to appreciate the need for data sharing and may participate in Taxonomic Expert Networks (TENs) as aggregators or do host *nomenclators*, provided technical expertise and support are available.

Nevertheless, even people responsible for helping users map names face a fragmented and sometimes confusing landscape of overlapping *taxonomic datasets* and *name-matching services* (Grenié et al. 2022). *Name-matching services* differ in features and can produce different results when matching the same pair of datasets. Clarifying terminology used to characterise service processes (e.g. “fuzzy matching”) and data labels (“scientific name”) is needed (Islam et al. 2024). We posit that *local users* also need guidance on which *taxonomic dataset* best suits their purpose; to provide this, we must define parameters for user needs and represent them as searchable *metadata* on the aggregator side.

## Taxonomic aggregators: datasets and services

To bring greater transparency to an otherwise confusing landscape, we have fixed the terminology we use in this article in the context of name matching. In Table 1, we defined *taxonomic aggregators* as online-accessible databases that host *taxonomic datasets*, i.e. structured lists of *scientific names*. Our focus here is on *taxonomic aggregators* that offer *name-matching services*. However, it proved useful to distinguish between the *taxonomic datasets* used by these services and the services themselves, even though these often go hand in hand. Some *taxonomic aggregators* do not offer their own *name-matching services* and some provide only an API (Application Programming Interface) for programmatic access without offering a readily available service for non-technical users. We also distinguish between *taxonomic aggregators* that function as dataset repositories and those that rely on a single aggregated *taxonomic dataset*.

### Taxonomic datasets

*Taxonomic datasets* typically present a hierarchical, tree-like taxonomy in which each taxon constitutes a node. Each *scientific name* is either assigned as the accepted name of a taxon or treated as a synonym (except in cases where the name exists, but cannot currently be resolved). As an exception from this definition, we include *nomenclators* as a special class of datasets. They systematically catalogue *scientific names* along with their authorship, publication date and references, nomenclatural status and (sometimes) type information and focus on nomenclatural accuracy, such as on correct spelling and nomenclatural validity rather than providing taxonomic opinion or classification.

Such taxonomic datasets aim to provide comprehensive coverage for particular organism groups, either globally or internationally (e.g. at a European scale). The Catalogue of Life monthly and annual editions are currently the only datasets attempting to unite all biota into a single global dataset (Hobern et al. 2021, Bánki et al. 2025). At the European level, PESI/EU-nomen (de Jong et al. 2015) serves a similar function for European taxa.

Data sources for the compilation of *taxonomic datasets* may include scientific literature, smaller *taxonomic aggregators*, data from publications or, in some cases, even original unpublished data. An important consideration regarding *taxonomic datasets* is the extent to which they are driven by and used by the respective taxonomic community.

The boundary between *local data* and the aggregator's *taxonomic dataset* may depend on context. For example, the Euro+Med PlantBase team, as a *local user*, has extensively used World Flora Online services for name matching to optimise EU-nomen processes (Müller and Güntsch 2025), with the aim of improving the *local data* and enabling the delivery of content data (primarily geographic distribution and common names) to the World Flora Online portal. Conversely, the Euro+Med taxonomic dataset (Euro+Med 2006-) forms part of the PESI/EU-nomen *taxonomic dataset* and *name-matching service*.

#### Categorising taxonomic datasets

To characterise datasets, a few basic parameters may be used:

- Taxonomic scope: Revision-scale (Genus, small family); Large *taxonomic group* (Plants, Fungi, Lichens, Butterflies, Mammals, Birds, ...); All organisms;
- Geographic scope: Local, State, National, Regional (supranational), Global;
- Thematic scope: pests, alien, Red List, marine species, …etc.;
- Completeness (for a given scope);
- Community scope: Broadly anchored and used dataset/database, Broadly used +/- individual contribution;
- Activity: Continuously updated; Static;
- Sustainability;
- Accessibility (open access);
- Linkability (persistent and resolvable identifiers).

Some of these properties are key criteria for users when selecting a dataset for their name-matching efforts.

### Name-matching services

We distinguish four main types of name-matching processes:

1. Direct use of the *name-matching service*: using the on-line user interface provided by the service (see Appendix 2);
2. Using third-party tools: Leveraging tools such as OpenRefine (an open-source tool for data cleaning and transformation), that access the *name-matching* services (see Mozzherin et al. (2024), also Appendix 3);
3. Using a web-accessible API or a programming package to access the *name-matching services* (see Appendix 4);
4. Using local tools: downloading the dataset and using local tools to perform the matching.

The choice of method depends mainly on the expected outcome, the number of records to be matched and the technical expertise available. Type 3 or 4 typically requires biodiversity-informatics expertise and even type 2 may not be attractive to non-technical users.

Data input depends on the chosen process. For type 1, a list of names may be pasted into a web interface or uploaded as a file in tabular form. For type 2, the *local data* must be loaded into the external tool. Type 3 and 4 processes depend on the form of local processing.

Results for process types 1 and 2 are provided as lists of exact matches and (for some services) lists of possible candidates. Some type 1 services offer interactive selection and acceptance of candidates as matches; this is also a feature of OpenRefine (type 2).

*Name-matching services* offered by *taxonomic aggregators* may rely on a single *taxonomic dataset* or the aggregator may act as a repository for multiple independent *taxonomic datasets* (COL-GBIF ChecklistBank, Global Names, TNRS, see Appendix 2). In the latter case, services can be configured to operate across all or selected datasets. When restricted to a single dataset, users can generally expect a taxonomically coherent outcome, otherwise they may use the matching to discover conflicting information. Existing *name-matching services* differ in their features and different services may even produce different results when matching the same two datasets, for example, when one service bases exact matches on *canonical names*, while another does not.

Beyond identifying exact matches between the input name and entries in the selected taxonomic dataset (matching in the strict sense, Hyam (2024)), a key functionality of *name-matching services* is their ability to suggest potential matches (candidates) for names that cannot be matched exactly. Various strategies address this challenge. For instance, some matching services restrict comparison to the *canonical name*, disregarding author citations or publication years. Another common strategy is fuzzy matching, which identifies similar names, based on minor character differences. Grenié et al. (2022) provide an overview of fuzzy-matching techniques and associated risks.

With respect to input and output for the *name-matching services*, we compiled a list of the input parameters and output fields of a number of services. From this, a kind of minimal common denominator for such services has been defined and a common vocabulary proposed (Hyam et al. 2025a). We also formulated a wish list of desired properties, based on use case studies, which may help to improve the services themselves or at least provide more transparency with regard to the methods used for matching and fuzzy matching (Berendsohn 2023b).

By analysing the challenges faced by *local users*, we first formulated a set of general recommendations across *taxonomic groups* (Appendix 1), providing an overview that already helps some *local users* identify suitable services and datasets. However, we recognised that this approach is insufficient, as more specific (and more general) user needs remain unmet. Additional guidance is provided through listings of the characteristics of available *name-matching services* (Appendix 2) and references to tools requiring technical expertise (Appendices 3 and 4). Yet, establishing a general workflow that systematically guides users towards an optimal dataset–service combination requires a more formalised approach. To this end, we sought to distill user requirements into a minimal set of questions that can be linked to *metadata* describing the services, datasets and their possible combinations. By prompting users to answer these questions, the workflow should help identify the most suitable dataset–service combination for their specific needs.

## A framework to harmonise name usage by name matching

### The workflow for mapping *local data* to *taxonomic datasets*

The core element of this workflow is the name-matching process. However, we observed significant variation in user requirements regarding the resulting mapping, i.e. the integration and linkage of *local data* with *taxonomic aggregators*. This variability also characterises the range of available *name-matching services* and their underlying *taxonomic datasets*, which may or may not meet specific user needs. Consequently, no single, universally applicable workflow exists.

Facilitating and streamlining the use of *name-matching services* may help to lower barriers and, thereby, increase linkages between taxonomic and non-taxonomic data that rely on names as the (initial) linking element.

There are six main steps in the workflow for mapping name usage in *local data* to *taxonomic datasets*, with steps 3-5 representing the name-matching process itself:

1. Defining the purpose of the mapping exercise;
2. Selecting the target aggregator (including the dataset and the associated name matching service);
3. Preparing the data: A list of names is required, which can be created from a textual table column or a database table. Each name should appear on a separate line. For file uploads, specific formats (column titles, delimiters) may be required;
4. Entering the data: This depends on the chosen type of checking process: pasting a list of names into a web interface, uploading a table or loading the data into a local tool;
5. Obtaining and interpreting the results: Online services and OpenRefine provide the results as lists of exact matches and possible candidates. Interpretation involves assessing these candidates and selecting the appropriate match;
6. Incorporating the results locally: This involves making local corrections, based on the matching results. Ideally, it also includes integrating the *taxonomic dataset's name ID* into the *local data*, enabling linkage and potential future interaction with the *taxonomic aggregator*.

We propose a simple set of user-orientated criteria to support step (1) and define *metadata* to support these criteria in the selection of relevant datasets and services for step (2).

Data preparation (step 3) largely consists of producing standardised name strings before a list of names can be used in the service. Some database systems offer such lists as exports in standard formats such as DwC (Darwin Core; Wieczorek et al. (2012)), ColDP (Catalogue of Life Data Package; Döring and Ower (2019)) or as flat-file exports (e.g. from the herbarium collection system JACQ; Rainer et al. (2023)). However, specific transformations may still be necessary to conform to the respective name-matching service. For example, the Catalogue of Life ChecklistBank tool requires standard column titles for uploaded tables. While DwC provides these directly, in JACQ the relevant field for the name is called NameAuthorYearString. Most services recognise only a single column - only in Catalogue of Life's ChecklistBank *canonical name* and author/year string may be provided separately, allowing users to obtain canonical and full-name matches in a single run. Some services allow users to interactively identify the column that contains names if it exists under another label in the uploaded table (e.g. WFO Plant List matching). A specific delimiter between column values (comma, tabulator) may be required or it may be detected automatically. We suggest that standardising these features would benefit users, beginning with a set of common input parameters and output fields (Hyam et al. 2025a), named in accordance with community standards.

The specific workflows implemented by each *name-matching service* (steps 3–5) are typically documented (some in more detail than others) by the service providers. We provide links to these resources to support users in effectively accessing and applying the services in Appendix 2.

The final part of the workflow (step 6) concerns how *local users* apply the matching results. This is highly dependent on users’ underlying motivations (see section on motivation above). Actions may range from correcting a misspelled name or fixing an erroneous classification, to incorporating a resolvable identifier from the *taxonomic dataset* or even to actively tracking changes in taxonomic concepts through a (yet-to-be-implemented) “Concept Subscription Service” (Hyam et al. 2025b).

To address the challenges posed by steps 1 and 2, we sought to demonstrate how an application, based on user requirements and metadata, can assist users in identifying a *name-matching service* suited to their particular needs. As a proof of concept, we developed a simple prototype (Fichtmüller et al. 2025).

### Characterising user requirements

We examine user requirements from the taxonomic name-matching perspective, as detailed under User motivation above, with the aim of defining *metadata* needed to match requirements with combinations of services and datasets. While some services may provide additional taxon information – such as geographic distribution, descriptions or images – name matching specifically to find such content is beyond this article's scope. The main selection criteria are classified as follows (combinations possible).

Organism group: Multiple or unknown groups OR one or more of the following: Animals, Plants, Algae, Fungi, Bluegreen Algae (Cyanobacteria), Bacteria, Virus (this list should be extended whenever a reasonably complete checklist for a large subgroup becomes available for name matching).

Geographic scope: Global; Continent or Macroregion; specific countries.

Thematic scope: Pests, invasives, marine, fossils etc. (marine species and fossils are available in the current prototype).

Number of names to be checked simultaneously: (choose one) up to 5000 or > 5000 (this is necessary because web interfaces for pasting names and certain file uploads restrict the number of names processed).

Target and purpose of name matching - I want to check (choose at least one):

- Orthography of *canonical name* (name without author / year);
- Orthography of full name (including author abbreviations and year);
- Acceptance (whether it is an accepted taxon name, a synonym of another name or taxonomically unresolved);
- Classification (list of higher taxa the name belongs to within the hierarchy of the aggregator's taxonomic dataset).

Data linkage requirements (choose one):

- I am not interested in identifiers and links for the name;
- I want a persistent identifier for the name in the aggregator's *taxonomic dataset*;
- I want a persistent resolvable identifier for that name;
- I want a persistent identifier for the taxon (accepted name) corresponding to the name I provide;
- I want a persistent resolvable identifier for that taxon.

Technical requirement - I want to (choose any):

- Enter or paste names into a web interface (usually limited number);
- Upload a list or table and receive the result as table or tables;
- Use OpenRefine as the name matching tool interface;
- Download an R package to perform name matching;
- Programme name matching using the aggregator's on-line API;
- Install the name matching service on my server;
- Download the data and perform matching within my own database.

### *Metadata* to the rescue

To identify the appropriate *taxonomic dataset* and *name-matching service* meeting user expectations, corresponding *metadata* for datasets and services must be defined.

***Metadata* for *taxonomic datasets***:

- Dataset ID – a unique (in future preferably resolvable) identifier for the entire dataset, used to relate to services;
- Dataset title and version: For implementation, this may be divided into short title, full title and version;
- Dataset homepage URL;
- Dataset description: As text and/or (preferably) with a URL to a page on the dataset's website;
- Content: Organism group(s), nomenclatural code(s); thematic scope; geographic scope (regions and countries, as a delimited list or individual flags);
- IDs and data linkage: has stable name ID*; has stable resolvable name ID*; name ID attribute (column) name; name ID resolved at URI; name ID resolves to taxon*; name ID resolves to name*; name ID caveats and notes;
- Downloads and searches: Full download from dataset website possible*; partial download possible*; download website URL; download formats; query URL;
- Access conditions: Open access*; licence or licence waiver; access conditions;
- Programming: Has API*; API-documentation URL.

* marks flags (Yes/No attributes).

***Metadata* for *name-matching services***:

- Service ID: A unique identifier for the service (to be used to relate to datasets);
- Service name, version, supporting organisation or institution;
- Service description: As text and/or (preferably) a URL to a page on the service website;
- Service user interface features: Has direct matching interface*; direct matching interface URL; direct matching restriction (number of name records); direct matching is interactive*; file upload for matching possible*; file upload URL; file upload format(s);
- Service processing features: Nomenclatural code (specification to avoid homonyms issues etc.); matching-process description; matching-process parameters; matching input to multiple datasets possible*; ignore terms (a list of common non-nomenclatural additions to the name, for example, “sp.”, “aff.”);
- Service output features: Output includes input columns*; output includes candidates*; output includes aggregator's name ID*; output file format(s);
- Programming: API with public access*; API documentation URL; API endpoint URL; is R-package*; OpenRefine reconciliation interface*; OpenRefine reconciliation interface URL; OpenRefine documentation URL; local installation possible*.

* marks flags (Yes/No attributes).

***Metadata* for dataset/service combination**:

- Caveats and notes;
- Full name checked*;
- Output includes: Taxonomic status*, nomenclatural status*, classification*, persistent ID*; persistent resolvable ID*;
- Dataset can be downloaded from service provider*.

* marks flags (Yes/No attributes).

### Proof of concept

Based on the *metadata* and user‑requirements described above, we have built a prototype web‑application that helps biodiversity‑data curators to select *name‑matching services* that best fit their particular needs. It is openly accessible at https://cetaf-eu.github.io/CheckMyName/. A complete description of its architecture and usage is provided by Fichtmüller et al. (2025) .

Tools that rely on *metadata* can become outdated rapidly because the underlying resources are continuously developed and their availability may change. One way to mitigate this problem is to enable community‑driven updating of the underlying tables (e.g. through a version‑controlled spreadsheet or a simple web‑form), but this often results in a rather patchy data coverage. Another, complementary approach is to involve data‑aggregators directly: if aggregators publish structured *metadata* about their *taxonomic datasets* and the *name‑matching services* they host, the guide could ingest this information automatically. At present, the *metadata* supplied by the Catalogue of Life’s ChecklistBank (see description on github) are mainly directed at document-type properties (dataset name, authors and editors, rights, abstract etc.), but also describe the taxonomic, geographic and temporal scope of each dataset. For the datasets stored, these fields may be empty; additional details must be extracted from the free‑text descriptions. Global Names, by contrast, provides only a textual description of each dataset (see the “i” icons under *Advanced options* at https://verifier.globalnames.org/).

A more robust solution would be for repositories such as ChecklistBank and Global Names to add a lightweight API or adopt a machine‑readable *metadata* schema that documents the properties of the *name‑matching service*. Single-dataset services could do the same. This would allow the guide to incorporate new resources without manual intervention.

### Outlook

The fundamental technical infrastructure required for the proposed framework already exists. The main obstacles that must be addressed before the framework can achieve widespread adoption are:

1. Data availability – Important resources (e.g. country‑level checklists) are still missing from *name‑matching services*, at least in some cases caused by access restrictions imposed by data contributors. This is limiting the guide’s relevance for many users;
2. Standardised *metadata* – No community‑agreed schema presently captures both the scope of a dataset and the characteristics of the associated matching service. Without such a standard, automatic aggregation and comparison of services remain difficult;
3. Ongoing curation – Keeping metadata up to date demands a modest, but continuous editorial effort and close coordination with data providers;
4. User awareness and uptake – Tools that are not widely known or perceived as useful are unlikely to be sustained.

Addressing these points will require:

1. Outreach to national biodiversity agencies and other data owners to encourage deposition of their checklists as open data in platforms such as Catalogue of Life's ChecklistBank or Global Names;
2. Development and endorsement of a community‑driven metadata standard (building on existing TDWG/GBIF specifications);
3. Implementation of a simple, web‑based interface for community contributions and routine updates;
4. Targeted promotion (e.g. tutorials, webinars, case‑study papers) to demonstrate the guide’s value to the different user communities.

## Conclusions

We have identified the matching process for scientific organism names as the core element for integrating datasets containing *scientific names* of organisms across the internet and for enabling science-based semantic linking of organism-related data. We found wide variation in user requirements as well as in the offered functionalities for this process. As a consequence, a single, universally applicable workflow does not exist, but rather a number of approaches that depend on the specific user interest. We posit that the basic technical mechanisms are essentially there, the necessary base data for taxon names and their interrelations are also becoming increasingly available (though with strong differences amongst organism groups and, in many cases, with questionable sustainability). Therefore, the biodiversity informatics infrastructure seems to be ready for a unified approach. Building on existing infrastructures to implement a user-centric approach to name matching can help to improve *data linkage* in biodiversity science and thus to address major problems in biodiversity research and conservation.

## Appendix 1: General recommendations for specific *taxonomic groups*

Some general recommendations can be given for specific *taxonomic groups.* Our focus here is on high-level organism groups (such as plants or animals) and, with respect to geographical representation, we focus on global coverage. A wider scope of sources is covered by Grenié & al. (2022).

Checking the names of animals

Although the zoological code of nomenclature (ICZN 1999) governs the application of names of animals, the vast zoological domain has resulted in multiple sub-communities treating specific taxa, some of them with comparatively huge species numbers, some of them of specific interest to the public (e.g. groups of vertebrates). Therefore, if users are interested specifically in, for example, fishes or birds, they are probably best served using bird or fish specific datasets. For example, name matching via Catalogue of Life’s ChecklistBank or Global Names Verifier is possible whenever the respective datasets have been deposited there, for example, FishBase (Froese and Pauly 2025), HBW and BirdLife International (2024) or the IOC World Bird List (Gill et al. 2025).

Checking the names of plants

Measured by species numbers, plants are a comparatively small group. However, even here specialised datasets exist, for example, for vascular plants, ferns and fern-allies and mosses (in the wide sense). Fungi and partly also algae, although mostly covered by the same code of nomenclature (in the past: the “botanical” code), are not considered plants here. Fortunately, botanical institutions have formed the World Flora Online (WFO) initiative (Wyse Jackson and Miller 2015, Borsch et al. 2020) that is incorporating various of these individual datasets (as the result of the work of Taxonomic Expert Networks). The WFO Plant List is increasingly becoming the normative source for a consensus classification of all plant names, closely collaborating with initiatives like the International Plant Name Index (IPNI 2000), the World Checklist of Vascular Plants (Govaerts et al. 2021) and Bryonames (Brinda and Atwood 2025). As it is providing stable and resolvable name IDs also for orthographic variants of names and other invalid designations used in a taxonomic context, we recommend using that dataset for plant name matching.

Checking the names of fungi

The mycological community has been building *scientific name* resources for decades and, since 1 January 2013, is requiring registration of new names (Turland et al. 2025). The registration identifier used by the three recognised registration centres for new names: IF (Index Fungorum 2009-), MycoBank (Crous et al. 2004) and Fungal Names (Wang et al. 2022) has also been applied to all pre-existing names, so that a consistent *name ID* exists for all fungal names. The repositories continuously synchronise their name holdings so that name matching against any of the repositories gives the same results in terms of name and identifier, with the exception of potentially different instances of taxon concepts and classifications of the names. The International Commission on the Taxonomy of Fungi (ICTF) is working on a consensus classification to be adopted by the three repositories. For name matching, Index Fungorum and Mycobank are available through Catalogue of Life’s ChecklistBank and Global Names Verifier; Fungal Names only through Global Names.

Checking the names of eukaryotic algae

AlgaeBase (Guiry and Guiry 2025) is integrated into the World Register of Marine Species (WoRMS Editorial Board 2025), with the exception of diatoms, which are managed in DiatomBase (Kociolek et al. 2025), also part of WoRMS/Aphia. Integration into WoRMS is made possible by the payment of an annual access fee by Lifewatch Flanders to support the content development within AlgaeBase. The dataset is not available for download through WoRMS due to licence conditions, but it can be queried through the WoRMS API. The AlgaeBase website does not offer a *name matching service*, but this is available in the context of the LifeWatch eLab TaxonMatch services. The service is currently restricted to exact matches due to the design of the AlgaeBase API. AlgaeBase is also available in an outdated version through Global Names Verifier. Algae are presently only incompletely covered by Catalogue of Life, because only data sources with either a CC-0 or CC-BY data license can be integrated. Further sources for algal names: Phycobank (Kusber et al. 2019) is a recognised repository for the registration of new names (not yet obligatory); AlgaTerra focuses on eukaryotic micro-algae; Index Nominum Algarum is a nomenclator comprising > 200,000 algal names compiled from index cards; the Catalogue of Diatom Names is a database not updated since 2011, but since 2018, it has been transformed into DiatomBase (see above). Of the latter resources, both DiatomBase (via its own Aphia matching service) and Index Nominum Algarum (through Catalogue of Life’s ChecklistBank) are currently accessible for name matching.

Checking the names of cyanobacteria (blue-green algae)

Cyanobacteria present a special case because they have traditionally been treated as algae and their name, thus, was governed by the International Code of Nomenclature for Algae, Fungi and Plants (ICNAFP, Turland et al. (2025)). However, they are prokaryotic organisms and thus, today, many cyanobacteria are named in accordance with the rules of the International Code of Nomenclature of Prokaryotes (ICNP, Parker et al. (2019)). This dual treatment may result in some names being valid under ICNAFP and not under ICNP. As a consequence, the choice of dataset (and related service) may depend on your research context. The LPSN (List of Prokaryotic names with Standing in Nomenclature) covers validly published names of cyanobacteria under the ICNP. This is most useful in a prokaryote-microbiological context and it is available in Catalogue of Life’s ChecklistBank and Global Names Verifier. AlgaeBase (see above) covers cyanobacterial names published under the ICNAFP in a phycological/botanical context, relevant, for example, for ecological research. NCBI Taxonomy (National Center for Biotechnology Information) includes (under bacteria) cyanobacterial names covered by ICNP and ICNAFP, but does not provide an accepted name for synonyms and does not claim to be an authoritative source for nomenclature or classification.

Checking names of Virus and Viroids

The internationally agreed dataset is the ICTV Master Species List (ICTV 2024) that provides the current taxonomic status of viral names and their classification. The website allows searching for individual viral names, but apparently, there is no bulk upload possible for name checking. No API is offered for machine access to the database at the ICTV website, but the dataset is included in Catalogue of Life’s ChecklistBank, Global Names Verifier and WoRMS (marine only).

## Appendix 2: Online *name-matching services*

Services may provide application programming interfaces (APIs) that often extend the possibilities of name matching or include further functions not provided by the interactive sites. This is also true for the R-packages listed in Grenié et al. (2022), see also Appendix 4. Undoubtedly these provide methods that can be highly useful for technically versed users; however, we refrained from testing anything that requires programming on the user’s side, with the sole exception of some simple coding used in the context of OpenRefine (see Appendix 3).

We distinguish between *name-matching services* that act upon repositories of *taxonomic datasets* and single-dataset services. The unfiltered user interface of the prototype https://cetaf-eu.github.io/CheckMyName/ provides a simplified table of service properties given in detail below.

### Repository services

Checklist Bank (GBIF & Catalogue of Life)

**Taxonomic scope**: All taxa or specific groups depending on the target dataset chosen. **Geographic scope**: Global or specific areas, depending on the target dataset chosen. **Software updated**: current (last checked 26 September 2025). **Codebase/Documentation**: https://www.checklistbank.org/about/API. **Data updated**: depending on target dataset. **Limitation**: Direct input of list limited to 6000 names (with file upload for asynchronous response not limited). **Local ID input returned**: YES. **Local Name input returned**: YES. **Aggregator name ID returned**: YES - in download only. **Interactive mode for partial matches**: NO. **OpenRefine reconciliation API**: NO (but for OpenRefine possible with REST services). **Other**: Login with GBIF account is recommended, required for file upload (self-registration at https://www.gbif.org/user/profile). **Other**: Offers the possibility to match datasets in repository against other such datasets. **Other**: API available

Global Names Verifier

**Taxonomic scope**: All taxa or specific groups depending on the target dataset chosen**. Geographic scope**: global (cross datasets or with global datasets) or restricted by choice of dataset. **Software updated**: current (last checked 26 September 2025)**. Codebase/Documentation**: https://github.com/gnames and https://resolver.globalnames.org/api**. Data updated**: Differs for stored datasets**. Limitation**: 5000 names, at least in interactive mode**. Local ID input returned**: NO**. Local Name input returned**: YES**. Aggregator name ID returned**: YES (may be a taxon ID)**. Interactive mode for partial matches**: NO**. OpenRefine reconciliation API**: YES, with step-by-step documentation including a video tutorial: https://github.com/gnames/gnverifier/wiki/OpenRefine-readme. **Other**: Offers a kind of query language that seems to be very flexible**. Other**: API and R package available

TNRS Taxonomic Name Resolution Service **also**: http://tnrs.iplantcollaborative.org/

**Taxonomic scope**: Plants, WFO; vascular plants WCVP - potentially more datasets could be included**. Geographic scope**: Global. **Software updated**: v. 5.0, 24 Feb 2021. **Codebase/Documentation**: https://github.com/ojalaquellueva/TNRSapi and https://github.com/iPlantCollaborativeOpenSource/TNRS/. **Data updated**: 2023/2024 [14 Dec 2025]**. Limitation**: Pasting 5000 names; API-processing unlimited (in batches of 5000). **Local ID input returned**: NO. **Local Name input returned**: YES. **Aggregator name ID returned**: NO**. Interactive mode for partial matches**: YES. **OpenRefine reconciliation API**: NO**. Other**: API and R package available.

### Single-dataset *name-matching services*

Australian Plant Name Index

**Taxonomic scope**: Plants. **Geographic scope**: Australia. **Software updated**: ? **Codebase/Documentation**: ? **Data updated**: Current. **Limitation**: result set limited to 100 matches, large uploads (1000 names) now blocked by Cloudflare [14 Dec 2025]: check with nearly 21,000 names ended in server error [May 2024]. **Local ID input returned**: NO. **Local Name input returned**: NO. **Aggregator name ID returned**: NO. **Interactive mode for partial matches**: NO. **OpenRefine reconciliation API**: NO. **Other**: APCalign: an R package workflow and app for aligning and updating flora names to the Australian Plant Census

GBIF Taxonomic Backbone

**Taxonomic scope**: All taxa. **Geographic scope**: global. **Software updated**: current. **Codebase/Documentation**: see Species API. **Data updated**: 28 August 2023 (no further updates, but will stay online; superseded by COL eXtended Release). **Limitation**: 6000 records. **Local ID input returned**: YES. **Local Name input returned**: YES. **Aggregator name ID returned**: NO. **Interactive mode for partial matches**: NO. **OpenRefine reconciliation API**: NO. **Other**: "Multi-taxonomy" mode available - allows to match against other, selected taxonomies (currently only the COL eXtended Release). **Other**: Matching Service including data is downloadable as a Docker image. **Other**: API available

International Plant Name Index (IPNI)

**Taxonomic scope**: Vascular plants (source: POWO; IPNI offered but not working). **Geographic scope**: Global. Software updated: ? **Codebase/Documentation** ? **Data updated**: current. **Limitation**: Not found - tested with 144.000 records. **Local ID input returned**: YES. **Local Name input returned**: YES. **Aggregator name ID returned**: YES: IPNI-LSID. **Interactive mode for partial matches**: NO. **OpenRefine reconciliation API**: YES - documentation: https://data1.kew.org/reconciliation/help.

PESI / EU-nomen

**Taxonomic scope**: All taxa. **Geographic scope**: Europe. **Software updated**: 2025. **Codebase/Documentation**: By reference to components used (Taxamatch algorithm and scientific name parser). **Data updated**: 2014 (update under way). **Limitation**: 1,000 names. **Local ID input returned**: YES (as an “extra” non-mapped column). **Local Name input returned**: YES. **Aggregator name ID returned**: YES (as provided by the primary aggregator). **Interactive mode for partial matches**: YES. **OpenRefine reconciliation API**: YES, via REST, see https://doi.org/10.3897/BDJ.2.e4221.

**TROPICOS (1986-)**

**Taxonomic scope**: Plants. **Geographic scope**: Global. **Software updated**: Name matching more than 10 years ago. **Codebase/Documentation**: No. **Data updated**: Current. **Limitation**: File size cannot exceed 2,048,576 KB. **Local ID input returned**: YES. **Local Name input returned**: YES. **Aggregator name ID returned**: YES: Tropicos-ID. **Interactive mode for partial matches**: NO. **OpenRefine reconciliation API**: NO.

World Flora Online WFO Plant List

**Taxonomic scope**: Plants. **Geographic scope**: Global. **Software updated**: ongoing October 2025 (not stated on website). **Codebase/Documentation**: GraphQL API, Name Matching REST API, Reconciliation API. **Data updated**: June 2025 (semi-annual edition). **Limitation**: Not found - tested with 144.000 records. **Local ID input returned**: YES. **Local Name input returned**: YES. **Aggregator name ID returned**: YES: WFO-ID. **Interactive mode for partial matches**: YES. **OpenRefine reconciliation API**: YES: https://list.worldfloraonline.org/reconcile_index.php. **Other**: Service can be installed as local copy. **Other**: R-Package World Flora - see https://cran.r-project.org/web/packages/WorldFlora/index.html. **Other**: API available.

WoRMS (World Register of Marine Species)

**Scope**: Marine species (global). **Software updated**: daily basis. **Codebase/Documentation**: in-house. **Data updated**: current. **Limitation**: limited to 1500 names. **Local ID input returned**: YES. **Local Name input returned**: YES. **Aggregator name ID returned**: YES: AphiaID. **Interactive mode for partial matches**: YES. **OpenRefine reconciliation API**: See Appendix 3. **Other**: API available.

## Appendix 3: OpenRefine reconciliation interfaces

OpenRefine is a powerful desktop application for cleaning and transforming data. A concise overview is available on Wikipedia. Mozzherin et al. (2024) present a compelling case for adopting OpenRefine reconciliation as a standard approach for *name-matching services*.

In the context of name matching, OpenRefine’s key feature is the ability to connect to external reconciliation services, i.e. APIs that match data entries against external sources. A reconciliation service can be used to match a column of *scientific names* in a table against the taxonomy provided by an aggregator service. The reconciliation services conform to the Reconciliation Service API, a specification developed by the W3C community.

Users normally install OpenRefine locally; installation packages are available for major operating systems, including a straightforward installer for Windows. Once installed, the desired name-matching service can be configured and connected to OpenRefine for repeated use.

To perform name matching, users load their tabular data into OpenRefine and initiate the reconciliation process on the column containing *scientific names*. In an additional new column, the service returns either exact matches or, when no exact match is found, a list of possible candidates (if any). Users can then review and select the appropriate match for each entry. Once reconciliation is complete, the enriched dataset including the new column containing the matched results can be exported for further use.

The following name-matching reconciliation services are available:

Global Names Verifier:

- Datasets: covering all datasets that have been imported into Global Names;
- Homepage (including an excellent step-by-step documentation for OpenRefine and a video tutorial): https://github.com/gnames/gnverifier/wiki/OpenRefine-readme;
- OpenRefine reconciliation endpoint: https://verifier.globalnames.org/api/v1/reconcile.

International Plant Name Index (IPNI):

- Dataset: IPNI;
- Homepage: https://data1.kew.org/reconciliation/about/IpniName;
- OpenRefine reconciliation endpoint: http://data1.kew.org/reconciliation/reconcile/IpniName.

Wikidata Reconciliation Service:

- Datasets: the entirety of Wikidata, but can be restricted to items that are instances of (P31) Taxon (Q16521) for better matches and faster processing;
- Homepage: https://wikidata.reconci.link/;
- OpenRefine reconciliation endpoint: https://wikidata.reconci.link/en/api.

World Flora Online:

- Dataset: WFO Plant List (latest semiannual version);
- Homepage: https://list.worldfloraonline.org/reconcile_index.php;
- OpenRefine reconciliation endpoint: https://list.worldfloraonline.org/reconcile.

Indirect access:

Catalogue of Life ChecklistBank:

- Developers recognised the need to implement a reconciliation API, but it is not yet available (see https://github.com/CatalogueOfLife/backend/issues/1265). Access can be implemented programmatically using the API.

WoRMS:

- Dataset: current;
- Homepage: https://github.com/SIB-Colombia/data-quality-open-refine;
- OpenRefine instructions: https://github.com/SIB-Colombia/data-quality-open-refine/blob/master/ValTaxonomicAPIWoRMS_ValTaxonomicaAPIWoRMS.txt.

## Appendix 4: Programming tools for name matching

### R-packages

R packages are prepackaged collections of code, data and documentation that extend the functionality of the R programming language. Grenié et al. (2022) cover this subject in detail, identifying 59 packages providing direct access to online taxonomic databases. They also point to an application (taxharmonizeexplorer) that should aid R-users to select tools and datasets. The website for the app lists and graphically depicts the relationship between taxonomic datasets and R-packages (as of end of November 2025, it covers 68 packages and 53 datasets). If this tool continues to be updated, it should be the primary source for R-programmers to identify useful packages for name matching and apply these in a workflow detailed in Grenié & al.’s paper.

### Application programming interfaces (APIs)

The following *name-matching services* offer a publicly accessible API:

- Catalogue of Life’s ChecklistBank Name Match - https://www.checklistbank.org/about/API;
- GBIF Species Matcher - https://techdocs.gbif.org/en/openapi/v1/species;
- Global Names Verifier - https://resolver.globalnames.org/api;
- Taxonomic Name Resolution Service (TNRS) - https://github.com/ojalaquellueva/TNRSapi?tab=readme-ov-file#readme;
- World Flora Online Plant List - https://list.worldfloraonline.org/matching_rest.php;
- WoRMS Taxon Match - https://www.marinespecies.org/aphia.php?p=webservice.

### Others

- Pytaxon: A Python software package for the identification and correction of errors in the taxonomic data of biodiversity species (Proença Neto and De Sousa 2025) - https://github.com/pytaxon/pytaxon-cli and https://zenodo.org/records/14538804.

## Figures and Tables

**Table 1.** Definition of terms as used in this article.

***Aggregator***	**See** *Taxonomic aggregator*
***Aggregator services***	Online accessible services (interactive or as endpoints for computer-to-computer communication) provided by *taxonomic aggregators*. Here focused on *name-matching services*.
***Associated data***	Data from a research or usage context that are linked to a *scientific name*.
***Canonical name***	A *scientific name* without the corresponding author or author-year citation.
***Data linkage***	Data linkage (or record linkage, Harron (2016)) is "the aggregation of data from different sources concerning the same entity or individual" (Vaccaro and Swerts 2022).
***Fuzzy matching***	A method to match scientific names that differ by some characters (Grenié et al. 2022).
***Local data***	A set of taxonomic names that a user wants to match against a *taxonomic dataset* using a *name-matching service*. The client side of name matching.
***Local user***	The person or team using a *name-matching service*.
***Metadata***	Data items that describe the content of datasets and the functionality of matching services, so that filters can be used to select them.
***Name-ID***	Name-Identifier: A number or code that is assigned to a scientific name in a taxonomic dataset, independent of its nomenclatural or taxonomic acceptance, for example, “wfo-0000481185”. A name-ID becomes resolvable if it includes or can be paired with a protocol to access an Internet resource (e.g. http://www.worldfloraonline.org/taxon/wfo-0000481185). A "persistent" identifier is designed to remain stable over time.
***Name matching***	The process of aligning a *scientific name* string with one in a *taxonomic dataset*, resulting in either an exact match or a candidate list of similar names (fuzzy matching).
***Name-matching service***	An *aggregator service* that allows to match scientific name strings from *local data* to one or more taxonomic datasets.
***Nomenclator***	A *taxonomic dataset* that is restricted to information about the *scientific name* itself, for example, its correct spelling or validity according to rules fixed in the international codes of nomenclature. The designation “taxonomic” for a dataset that is essentially only referring to nomenclature is used here for convenience.
***Scientific name***	A name formed in accordance with the international rules of nomenclature (for animals, for algae, fungi and plants, for bacteria and for viruses different codes apply).
***Synonym***	A *scientific name* that is not accepted for a taxon, but recognised as belonging to one. Non-acceptance may have nomenclatural reasons (species now in a different genus, older name has same type specimen, name was not adhering to the rules when published). Or a name may have been the name of a taxon that taxonomists now consider to fall into the circumscription of another (accepted) taxon.
***Taxonomic aggregator***	Here: Online accessible databases that compile taxonomic information from multiple sources into a *taxonomic dataset* or act as a repository hosting *taxonomic datasets* from multiple sources.
***Taxonomic dataset***	Here: structured lists of *scientific names* hosted by a *taxonomic aggregator*. With the exception of *nomenclators*, they typically present a hierarchical, tree-like taxonomy (classification) in which each taxon represents a node and each *scientific name* is either assigned as the accepted name of a taxon or treated as a synonym (except in cases where the name exists, but cannot currently be resolved). This term largely corresponds to the term Taxonomic Database used by Grenié et al. 2022.
***Taxonomic group***	A broad term to describe a group of taxa or taxonomic ranks at which people work (Grenié et al. 2022), for example, plants, fungi, animals.
